# Definition of a Skp2-c-Myc Pathway to Expand Human Beta-cells

**DOI:** 10.1038/srep28461

**Published:** 2016-07-06

**Authors:** Shiwani Tiwari, Chris Roel, Mansoor Tanwir, Rachel Wills, Nidhi Perianayagam, Peng Wang, Nathalie M. Fiaschi-Taesch

**Affiliations:** 1 Diabetes Obesity and Metabolism Institute, Icahn School of Medicine at Mount Sinai, New York, NY, 10029, USA; 2 The Division of Endocrinology, University of Pittsburgh School of Medicine, Pittsburgh, PA, 15213, USA.

## Abstract

Type 2 diabetes (T2D) is characterized by insulin resistance and reduced functional β-cell mass. Developmental differences, failure of adaptive expansion and loss of β-cells via β-cell death or de-differentiation have emerged as the possible causes of this reduced β-cell mass. We hypothesized that the proliferative response to mitogens of human β-cells from T2D donors is reduced, and that this might contribute to the development and progression of T2D. Here, we demonstrate that the proliferative response of human β-cells from T2D donors in response to cdk6 and cyclin D3 is indeed dramatically impaired. We show that this is accompanied by increased nuclear abundance of the cell cycle inhibitor, p27^kip1^. Increasing nuclear abundance of p27^kip1^ by adenoviral delivery decreases the proliferative response of β-cells from non-diabetic donors, mimicking T2D β-cells. However, while both p27^kip1^ gene silencing and downregulation by Skp2 overexpression increased similarly the proliferative response of human β-cells, only Skp2 was capable of inducing a significant human β-cell expansion. Skp2 was also able to double the proliferative response of T2D β-cells. These studies define c-Myc as a central Skp2 target for the induction of cell cycle entry, expansion and regeneration of human T2D β-cells.

T2D (T2D) has traditionally been regarded to be the result of insulin resistance in liver, skeletal muscle and adipose tissue^1^,^2^,^3^. Recently, autopsy and genome-wide association studies (GWAS) suggest that it is also associated with β-cell deficiency and dysfunction^4^,^5^,^6^,^7^,^8^,^9^,^10^. The major factor associated with T2D is obesity, although not all obese subjects become diabetic^1^,^3^,^10^. In autopsy studies, patients with T2D display a reduced β-cell mass as compared to non-diabetic patients with comparable BMI. In contrast, β-cell mass is increased in non-diabetic obese subjects as compared to lean subjects^8^,^11^,^12^. In rodents, β-cell expansion in obesity models is associated with replication of endogenous β-cells^1^,^3^. However, there is little evidence for β-cell replication in human obesity or T2D.

In humans, understanding how the β-cell mass evolves during insulin resistance and the development of T2D is challenging due to the limitations of autopsy studies. Studies in children and young adults suggest that it is possible that some people accrue lower than average β-cell mass during their first years of development^13^,^14^. These individuals would thus require greater expansion of β-cell mass in response to insulin resistance. Indeed, β-cell mass is primarily established during the first years after birth and is highly variable among children and young adults^13^,^14^. A second possibility is that if β-cell expansion can occur in adults, some individuals may not expand their β-cell mass as effectively as others in response to obesity and insulin resistance. A third possibility is that β-cell death and/or dedifferentiation may be more prevalent in some individuals, leading to the emergence of T2D. Finally, it is likely that combinations of the above occur. In any case, the failure of β-cells to adapt to insulin resistance seems to be central to the development of T2D, whether due to reduced β-cell proliferative response, and/or increased β-cell death, and/or loss of β-cell function and de-differentiation.

A number of studies have linked the deregulation of cell cycle genes in β-cells with the development of T2D. In GWAS studies, T2D susceptibility loci have been identified in or near cell cycle genes^6^,^7^. In mouse genetic studies, the cell cycle inhibitor, p27^kip1^, has been linked to the development of T2D. For example, p27^KIP1^ progressively accumulates in the nuclei of pancreatic β-cells in T2D mouse models which lack either the insulin receptor substrate 2 (IRS2), or the leptin receptor^15^. In these two models of T2D, the genetic knockout of p27^kip1^ reduces the hyperglycemia, increases β-cell mass and maintains hyperinsulinemia, predominantly via β-cell proliferation. In addition, p27^kip1^ mRNA is increased in islets from human T2D donors as compared to non-diabetic donors^16^.

p27^kip1^ may be either an activator or inhibitor of cell cycle progression. In rodent β-cells, p27^kip1^ has been shown to be a cell cycle inhibitor^17^,^18^,^19^. However, in other cell types, p27^kip1^ has also been shown to act as an activator of cell cycle. By facilitating the formation and stabilizing the complex formed between D-cyclins and cdk4 or cdk6, p27^kip1^ acts as a chaperone for the assembly and nuclear translocation of the complex^20^. This leads to an activation of cell cycle entry. With regards to human β-cells, p27^kip1^ is known to be expressed in whole human islets^21^ and in human β-cells, mostly in their cytoplasm^22^,^23^. […]. The precise role of p27^kip1^ in regulating β-cell mass and proliferation is not known in humans.

p27^kip1^ expression is mostly regulated post-transcriptionaly by poly-ubiquitinylation and proteosomal degradation. The S-phase kinase-associated protein 2 (Skp2), a component of the SCF (Skp1-Cullin 1-F-box) E3 ubiquitin-ligase complex, has been shown to be the major p27^kip1^ -ubiquitin ligase. Although p27^kip1^ is a critical target of Skp2, many additional substrates of Skp2 has been identified. Many of these proteins, such as p21^cip^, p57^kip2^, E2F1, MEF, p130 Tob1, cyclin D1, cyclin E, Smad4 and c-myc are cell cycle regulators^24^. c-myc is unique among Skp2 targets, since Skp2 induces not only its ubiquitinylation, but also increases its transcriptional activity^25^,^26^,^27^.

While several studies have focused on β-cell loss of function and/or de-differentiation and/or susceptibility to cell death in T2D, no prior study has examined the possibility that the proliferative response of human β-cell from T2D donors is impaired. We hypothesized that this is the case. We further hypothesized that abundance of p27^kip1^ may be elevated in the nuclei of human T2D β-cells, limiting their response to mitogens. Finally, we hypothesized that the downregulation of p27^kip1^ might lead to increased proliferative response of human β-cells.

## Methods

### Human cadaveric islets

Human islets were provided by the NIH- and JDRF-supported Integrated Islet Distribution Program (iidp.coh.org), Prodo Lab (Irvine, CA) and the University of Alberta (Edmonton, Alberta, Canada). One hundred and six different cadaveric preparations from non-diabetic donors and six from T2D donors were used for these studies. The means age of the donors was 44.5 years ± 1.2 with a BMI of 30.9 ± 0.6 kg/M^2^ for the non-diabetic donors and 54.0 years ± 3 with a BMI of 29.3 ± 2.4 kg/M^2^ for the T2D donors. Upon arrival, islets were incubated in RPMI medium (Gibco, Grand Isle, NY) containing 5.5 mM glucose, 1% penicillin and streptomycin with 10% fetal bovine serum until they were utilized for experiments. The use of human cadaver islets was approved by and in accordance with University of Pittsburgh School of Medicine and the Icahn School of Medicine at Mount Sinai Institutional Review Boards.

### Adenovirus production and transduction

Adenoviruses, all under control of the CMV promoter, were prepared using human cDNAs encoding human cdk6, human cyclins D3 and human c-Myc as described previously^22^,^23^,^28^,^29^. Ad.human Skp2 was purchased from Vector Biolabs (Philadelphia, PA). Dispersed islets on coverslips were transduced with either Ad.LacZ or relevant control adenoviruses for two hours, cultured for 72 hours as described in the Figures, and immunolabeled as described below.

### Islet Cell Dispersion and Immunocytochemistry

Human islets were aliquoted into 400 IEQ and washed twice in PBS and dispersed, transduced, fixed and immunolabelled as previously described^18^,^19^,^24^,^25^. Primary antisera and secondary antisera are described in Supplemental Table 1.

### Immunoblotting

Islet extracts were resolved using 10% or 12% SDS-PAGE, and transferred to Immobilon-P membranes (Millipore Inc., Bedford, MA). Primary antisera and secondary antisera are described in Supplemental Table 1. Each experiment shown is representative of 3–6 human islet preparations.

### Assessment of β-cell mass *in vitro*

Human islets were dispersed as described previously^18^,^19^,^24^,^25^ and transduced with either Ad.LacZ or Ad.cdk6 + Ad.cyclinD3 or Ad.cdk6 + Ad.cyclinD3 + Ad.Skp2 or Ad.cdk6 + Ad.cyclinD3 + Ad.shp27^kip1^ as described previously. Ten thousand transduced islets cells from each group were plated in a high-content imaging glass bottom 96 well plate (Corning, Corning, NY), coated with poly-D lysine (Sigma-Aldrich, St Louis, MO). Five, 10, 15 and 20 thousand untransduced dispersed human islet cells were plated as controls. One day later, 50 ul of Matrigel (Corning, Corning, NY) was added to each well, and cells were fixed five days after plating. Cells were then immunolabelled for insulin and DAPI as previously described^18^,^19^,^24^,^25^. The β-cell mass was assessed as previously described^28^.

### Real-Time PCR

Human islets were dispersed as described previously and transduced with either Ad.LacZ or Ad.cdk6 + Ad.cyclinD3 or Ad.cdk6 + Ad.cyclinD3 + Ad.Skp2 or Ad.cdk6 + Ad.cyclinD3 + Ad.c-Myc as described previously. Three days after transduction, islets cells were harvested and mRNA extracted using the Absolutely RNA miniprep purification kit (Agilent Technologie, Santa Clara, CA) following the manufacturer’s instructions. cDNA and quantitative real-time PCR were performed as previously described^29^. Primers sequences are in Supplemental Table 2.

### Statistics

**Figure 1:**
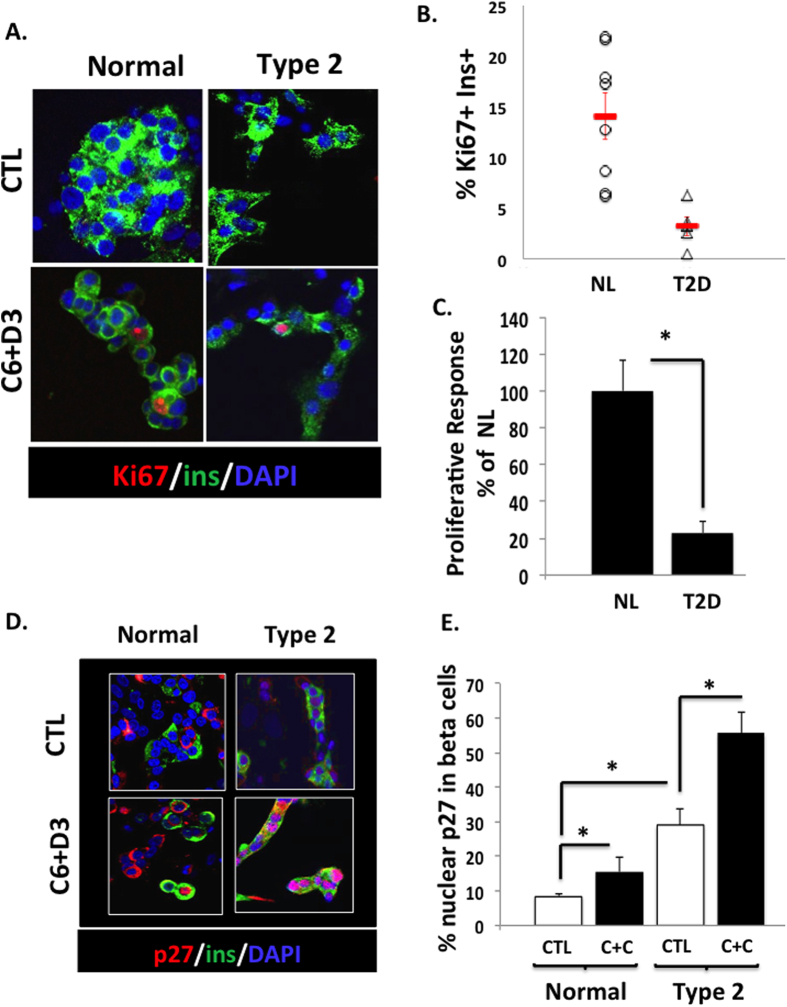
Proliferative response and p27^kip1Cip/Kip^ expression in T2D human β-cells. (**A**) Dispersed human islets from normal (Normal) or T2D (Type 2) donors were transduced with control adenovirus (“CTL”) or with Ad.cdk6 and Ad.cyclin D3 (“C6 + D3”). Immunolabelling for Ki67 is shown in red, insulin in green, and DAPI in blue. (**B**) Scattered plot of % of Ki67+/insulin + cells in response to cdk6 and cyclin D3 in non-diabetic β-cells (NL) (n = 8) and in β-cells from T2D donors (T2D) (n = 5). (**C**) Quantitation of % of Ki67+/insulin + cells. Bars indicate mean ± SEM. (**D**) Dispersed human islets from normal (Normal) (n = 7) or T2D (Type 2) (n = 6) donors were transduced with control adenovirus (“CTL”) or with Ad.cdk6 and Ad.cyclin D3 (“C6 + D3”). Immunolabelling for p27^kip1cip/kip^ is shown in red, and insulin in green. (**E**) Quantification of data in C at 72 hours after cdk6 + cyclin D3 transduction. Bars indicate mean ± SEM of the % nuclear localization of the p27^kip1^ shown in insulin+ (beta) cells.

**Figure 2:**
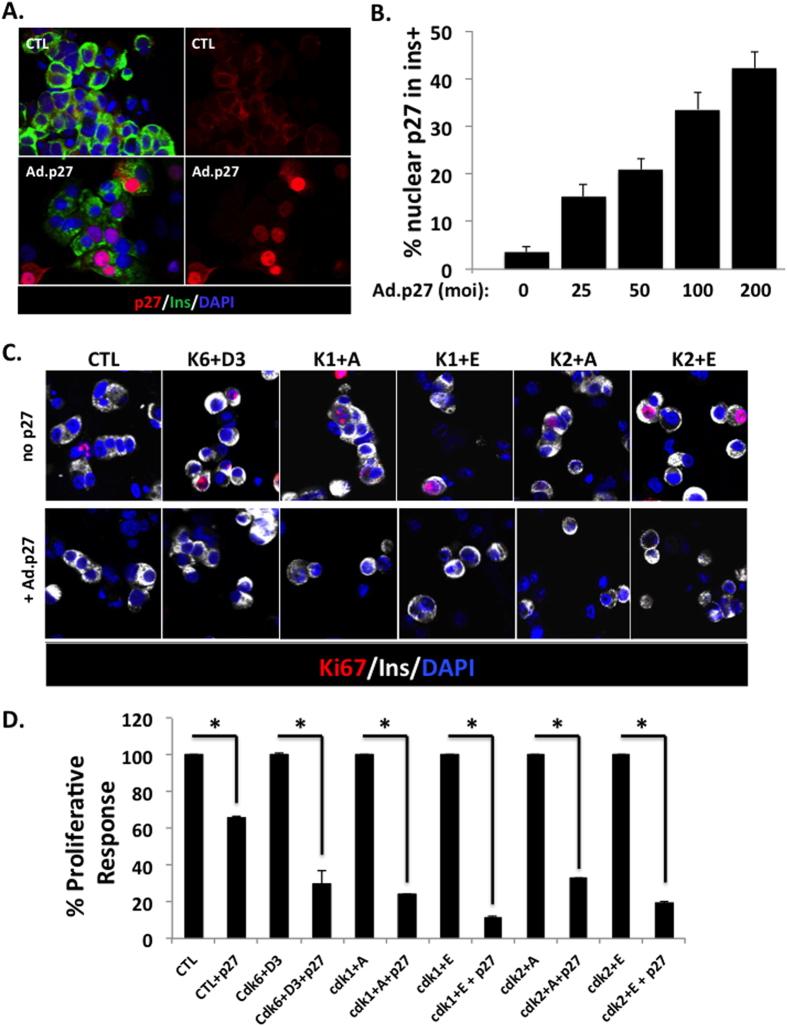
Effect of p27^kip1^ overexpression on human β-cell proliferation. **(A**) Dispersed human islets from normal donors (n = 5) were transduced with control adenovirus (CTL) or with of Ad.p27^kip1^. Immunolabelling for p27^kip1^ is shown in red, insulin in green, and DAPI in blue. (**B**) Quantitation of the % nuclear localization of the p27^kip1^ shown in insulin+ (beta) cells with increasing doses of adenovirus. Bars indicate mean ± SEM. (**C**) Dispersed human islets from normal donors (n = 3–5) were transduced with control adenovirus (CTL) or with cdk6 and cyclin D3 (K6 + D3), or cdk1 and cyclin A (K1 + A), or cdk1 and cyclin E (K1 + E), or cdk2 and cyclin A (K2 + A), or cdk2 and cyclin E (K2 + E), without (top panels) or with (bottom panels) Ad.p27^kip1^. Immunolabelling for Ki67 is shown in red, insulin in white, and DAPI in blue. (**D**) Quantification of data in C at 72 hours after transduction. Bars indicate mean ± SEM of the % of the proliferation in each condition without Ad.p27^kip1^.

**Figure 3:**
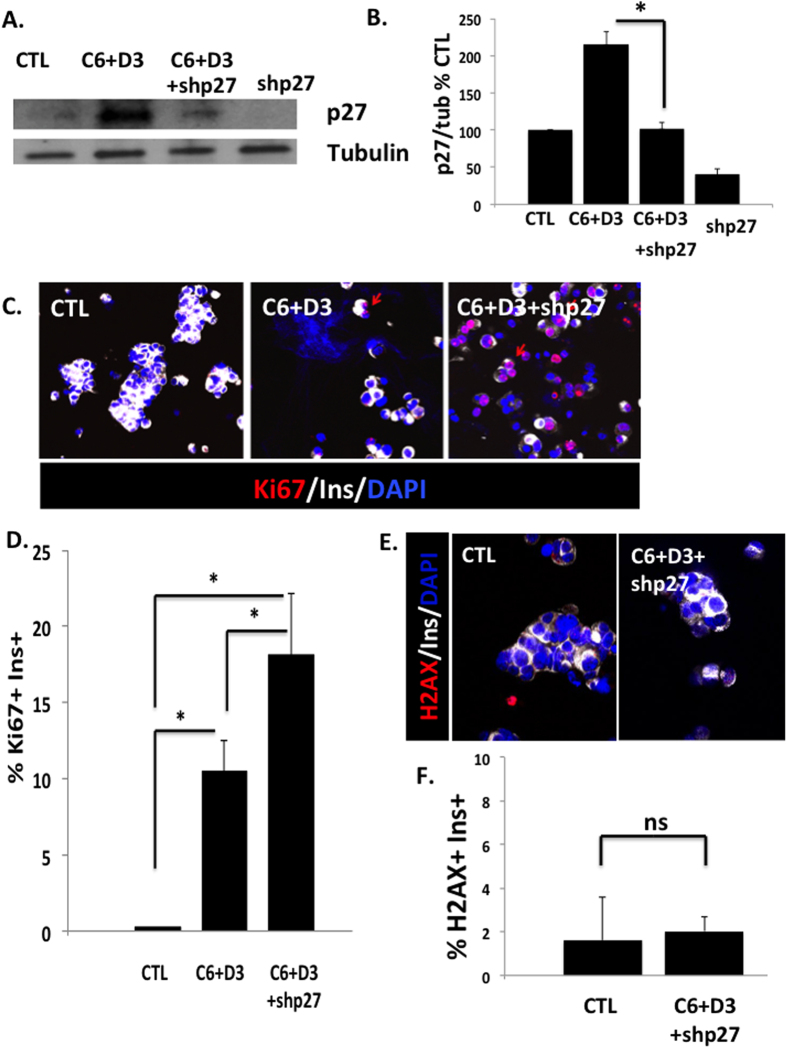
P27^KIP1^ decreases the proliferative response of human β-cells. (**A**) Representative immunoblot of human islets transduced with a control adenovirus (CTL) or with Ad.cdk6 and cyclin D3 (C6 + D3), or with Ad.cdk6 and cyclin D3 with shRNA to silence p27^kip1^ (C6 + D3 + shp27^kip1^), or with shRNA to silence p27^kip1^ (shp27^kip1^). (**B**) Densitometric analysis of p27^kip1^ immunoblots. Experiments were repeated five times. *p < 0.05. (**C**) Dispersed human islets from normal donors were transduced with control adenovirus (“CTL”) or with Ad.cdk6 and Ad.cyclin D3 (“C6 + D3”) or with Ad.cdk6 and cyclin D3 with shRNA to silence p27^kip1^ (C6 + D3 + shp27^kip1^). Immunolabelling for Ki67 is shown in red, insulin in white, and DAPI in blue. (**D**) Quantification of data in C at 72 hours after transduction. Bars indicate mean ± SEM of the % of the proliferation with cdk6 and cyclin D3 (n = 5). (**E**) Dispersed human islets from normal donors were transduced with control adenovirus (“CTL”) or with Ad.cdk6 and cyclin D3 with shRNA to silence p27^kip1^ (C6 + D3 + shp27^kip1^). Immunolabelling for γ-phospho-H2AX (H2AX) is shown in red, insulin in white, and DAPI in blue. (**F**) Quantification of H2AX immunolabelling at 72 hours after transduction (n = 5). Bars indicate mean ± SEM of the % of H2AX positive β-cells.

**Figure 4:**
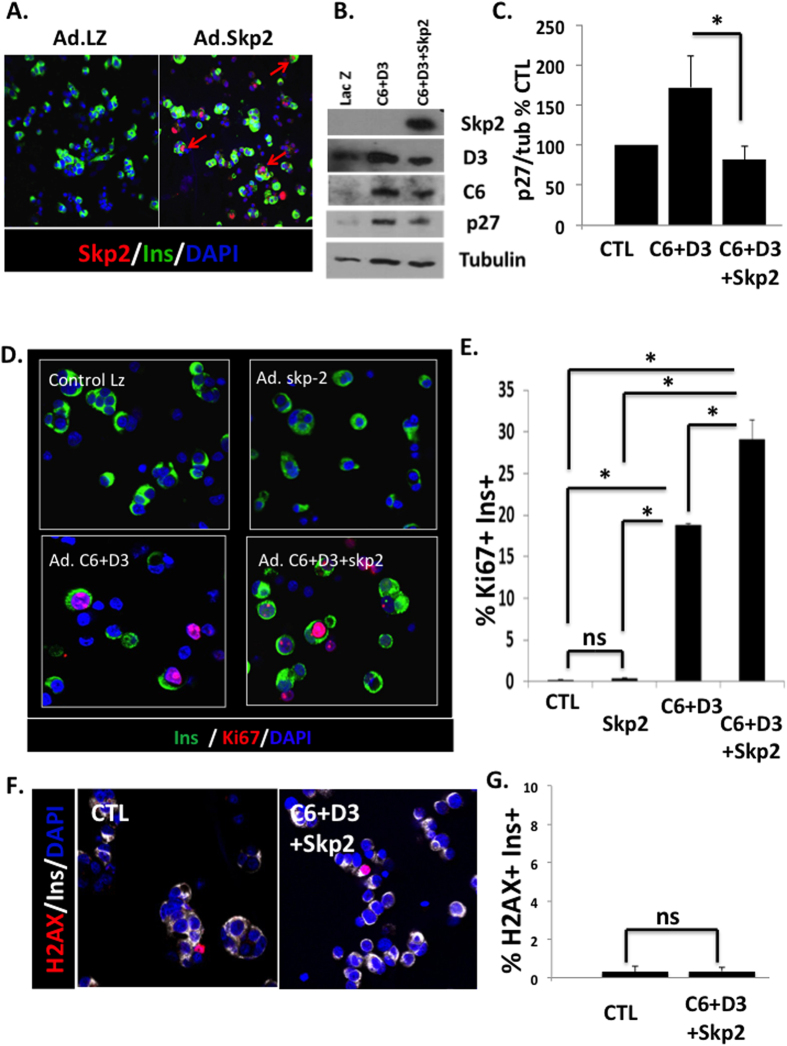
Skp2 reduces p27^kip1^ expression and enhances the proliferative response of human β-cells. (**A**) Dispersed human islets from normal donors were transduced with control adenovirus (“CTL”) or with Ad.Skp2. Immunolabelling for Skp2 is shown in red, insulin in green, and DAPI in blue (n = 3). (**B**) Immunoblot of human islets transduced with a control adenovirus (Lac Z) or with Ad.cdk6 and cyclin D3 (C6 + D3), or with Ad.cdk6 and cyclin D3 and Skp2 (C6 + D3 + skp2). (**C**) Densitometric analysis of p27^kip1^ immunoblots. Experiments were repeated three to five times. *p < 0.05. (**D**) Dispersed human islets from normal donors were transduced with control adenovirus (“CTL”) or with Ad.cdk6 and cyclin D3 (C6 + D3), or with Ad.cdk6 and cyclin D3 and Skp2 (C6 + D3 + skp2). Immunolabelling for Ki67 is shown in red, insulin in green, and DAPI in blue. (**E**) Quantification of data in C at 72 hours after transduction. Bars indicate mean ± SEM of the % of Ki67 in insulin + cells, (n = 8). (**F**) Dispersed human islets from normal donors were transduced with control adenovirus (“CTL”) or with Ad.cdk6 and cyclin D3 and Skp2 (C6 + D3 + Skp2). Immunolabelling for γ-phospho-H2AX (H2AX) is shown in red, insulin in white, and DAPI in blue. (**G**) Quantification of H2AX immunolabelling at 72 hours after transduction. Bars indicate mean ± SEM of the % of H2AX positive β-cells (n = 6).

**Figure 5:**
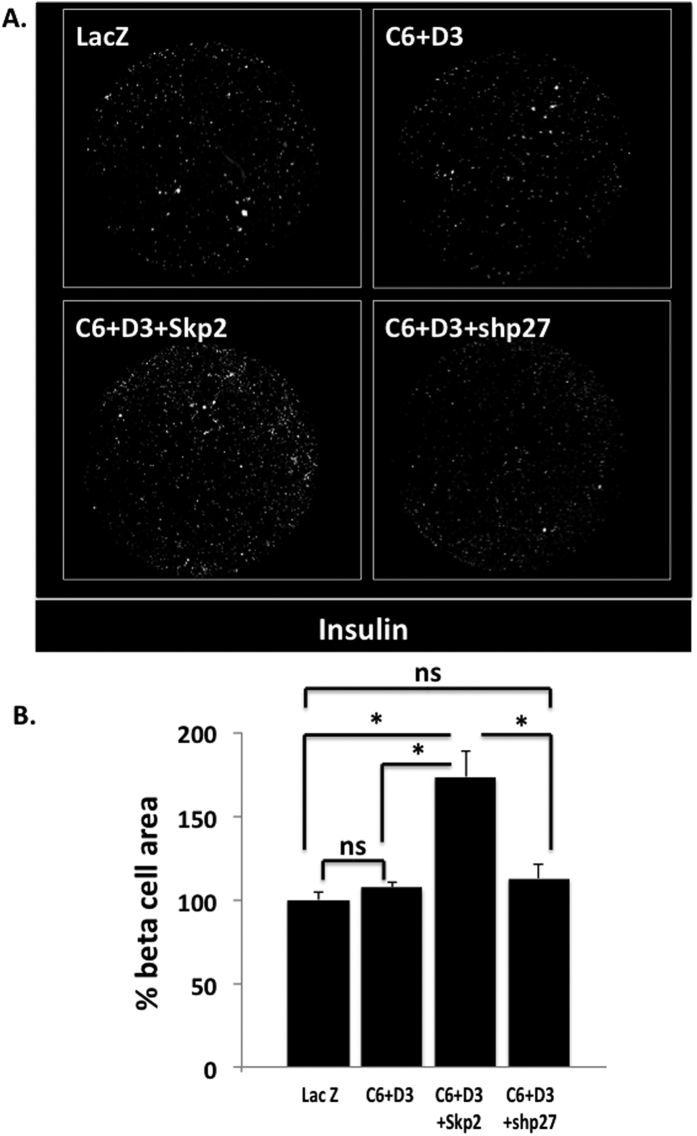
Skp2, but not p27^kip1^ silencing, induces the expansion of human β-cells. **(A**) Representative picture-montages covering the entire wells of high imaging content 96-well plates plated with dispersed human islets cells. Dispersed human islets (ten thousand islet cells), transduced with Ad.LacZ (LacZ), Ad.cdk6 + cyclin D3 (K6 + D3), or Ad.cdk6 + cyclin D3 + Skp2 (K6 + D3 + Skp2) or Ad.cdk6 + cyclin D3 + shp27^kip1^ (K6 + D3 + shp27^kip1^) were plated and fixed five days after plating. Islet cells were immunolabelled for insulin (shown in grey). (**B**) Quantitation of the insulin staining area as a percentage of the control ten thousand islet cells transduced with LacZ. (n = 8).

**Figure 6:**
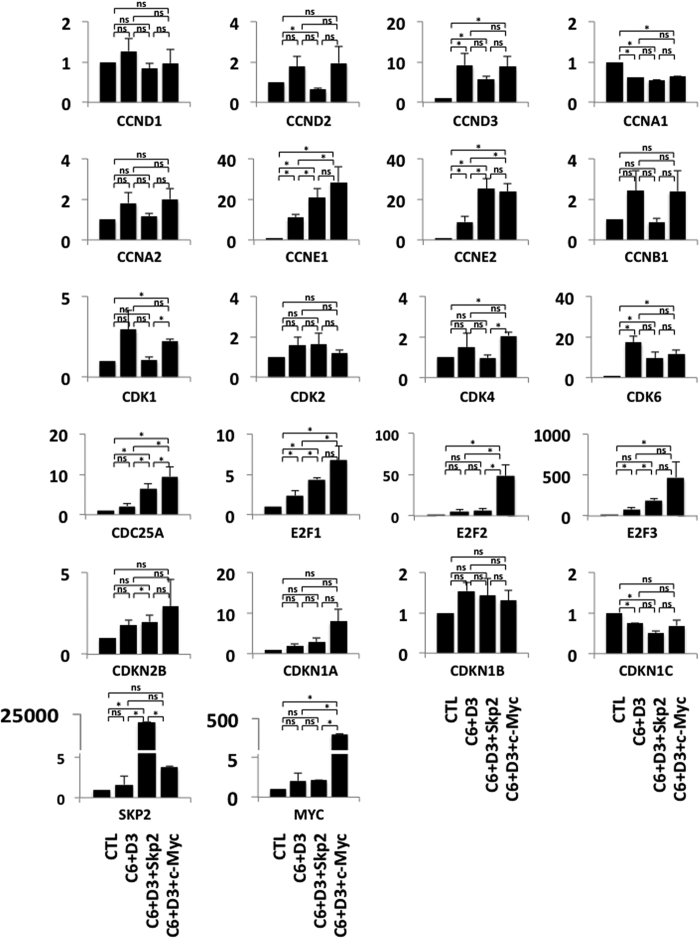
Skp2 induces c-Myc downstream target cell cycle genes. Dispersed human islets from normal donors were transduced with control adenovirus (“CTL”) or with Ad.cdk6 and cyclin D3 (C6 + D3), or with Ad.cdk6 and cyclin D3 and Skp2 (C6 + D3 + skp2) or with Ad.cdk6 and cyclin D3 and c-Myc (C6 + D3 + c-Myc). The transduced islet cells were then harvested three days after transduction, and the mRNA was extracted. Real-time qPCR was performed. Data for the D-, A, E- and B-cyclins, cdk1, 2, 4 and 6, cdc25a, E2F1, 2 and 3, p15, p21, p27^kip1^, p57, Skp2 and c-Myc are shown. Experiments were repeated on five to seven human islet preps.

**Figure 7:**
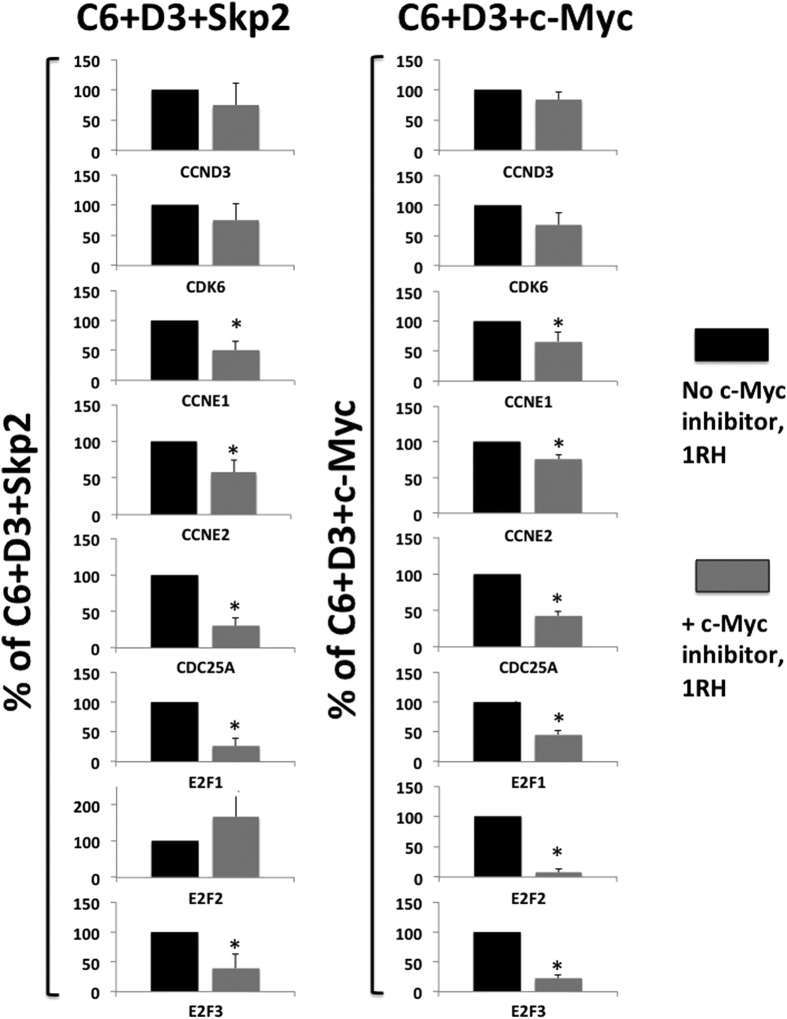
Skp2 induces cell cycle genes in a c-Myc-dependent manner. Dispersed human islets from normal donors were transduced with Ad.cdk6 and cyclin D3 and Skp2 (C6 + D3 + skp2, left panels) or with Ad.cdk6 and cyclin D3 and c-Myc (C6 + D3 + c-Myc, right panels) without (black bars) or with (grey bars) the c-Myc inhibitor 1RH. The transduced islet cells were then harvested three days after transduction, and the mRNA was extracted. Real-time qPCR was performed. Data for the cyclin D3, E1, E2, cdk6, cdc25a, E2F1, E2F2 and E2F3 are shown. Experiments were repeated on four to five human islet preps.

**Figure 8:**
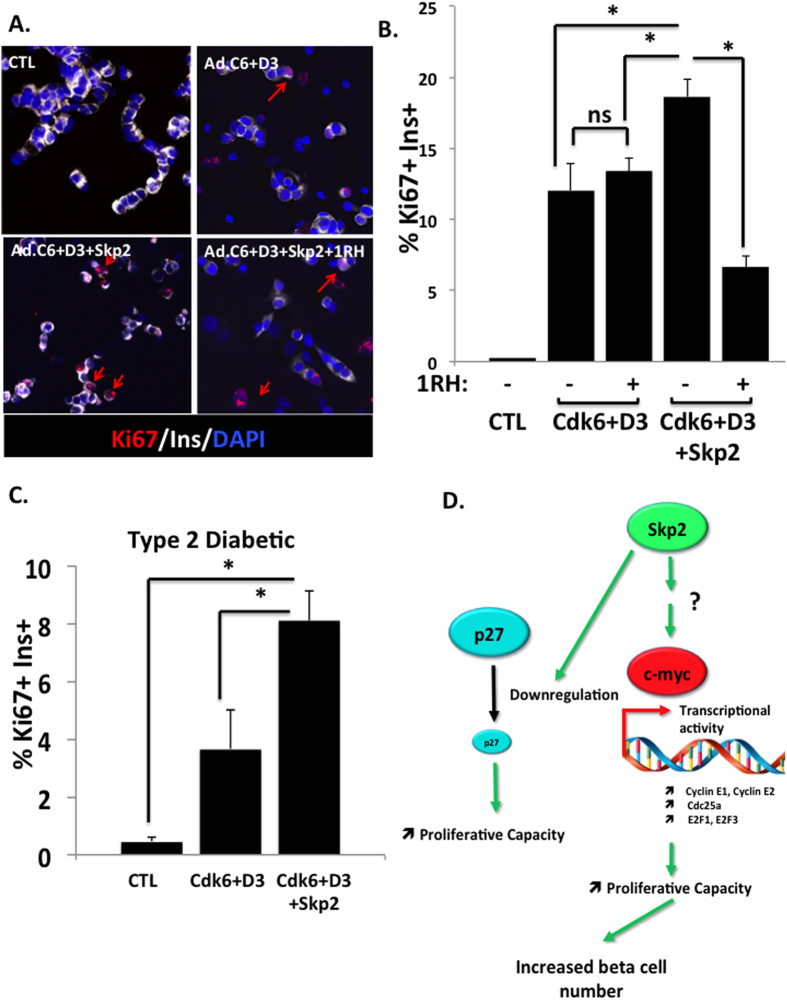
Skp2 enhances the proliferative response of human β-cells in a c-Myc dependent manner and increases the proliferative response of T2D β-cells. (**A**) Dispersed human islets from normal donors were transduced with control adenovirus (“CTL”) or with Ad.cdk6 and cyclin D3 (C6 + D3), or with Ad.cdk6 and cyclin D3 and Skp2 (C6 + D3 + skp2) and treated with or without the c-Myc inhibitor 1RH. Immunolabeling for Ki67 is shown in red, insulin in white, and DAPI in blue. Experiments were repeated on at least four human islet preps. (**B**) Quantification of data in C at 72 hours after transduction. Bars indicate mean ± SEM of the % of the proliferation with cdk6 and cyclin D3. (**C**) Dispersed human islets from T2D donors were transduced with control adenovirus (“CTL”) or with Ad.cdk6 and cyclin D3 (C6 + D3), or with Ad.cdk6 and cyclin D3 and Skp2 (C6 + D3 + skp2). Quantification of the % of Ki67 in insulin + cells at 72 hours after transduction. Bars indicate mean ± SEM of the % of Ki67 in insulin + cells. Experiments were repeated on at least three human islet preps from T2D donors. (**D**) Schematic cartoon of the actions of Skp2 in human β-cells. Skp2 reduces the expression of p27^kip1^. The reduction of p27^kip1^ expression alone leads to an increased proliferative capacity, but not an increased in β-cells number. Skp2 also increases, directly or indirectly (as indicated by multiple arrows and a question mark), the transcriptional activity of c-Myc, inducing the expression of cyclin E1, E2, cdc25a, E2F1 and E2F3, therefore leading to an increased proliferative capacity and expansion of human β-cells *in vitro*.

Statistical analysis for Figs 1E, 2D, 3C, 4D, 5B, 6 was performed using one-way ANOVA for repeated variable with Bonferroni’s post-hoc correction. Student’s paired, two-tailed T-test was employed in Figs 1C, 3D, 4E and 7. A one-way ANOVA for repeated variable with Bonferroni’s post-hoc correction was performed for Fig 8B,C. All values are expressed as means ± SEM. “p” values < 0.05 were considered significant.

## Results

### The proliferative response of human T2D β-cells is reduced

Since β-cell mass is diminished in T2D individuals^8^, we explored the proliferative response of β-cells from T2D donors by overexpressing cdk6 and cyclin D3 in human dispersed islets. In this system, 14.1 ± 2.3% of normal human β-cells were labeled with Ki67 (Fig. 1). This robust response permits the proliferative response to be easily assessed. As shown in Fig. 1A–C, the proliferative response of T2D human β-cells was dramatically reduced by 80% as compared to non-diabetic β-cells.

### p27^kip1^ nuclear abundance is increased in β-cells from T2D donors

Since p27^kip1^ is an important cell cycle inhibitor in rodent β-cells, and since p27^kip1^ overexpression is linked to T2D in rodent models, we questioned whether the reduction of proliferative response in T2D human β-cells correlates with an increased level of nuclear p27^kip1^. As shown in Fig. 1D,E, in human β-cells from normal and T2D donors, p27^KIP1^ was detected more often in the nuclei of T2D β-cells as compared to normal β-cells, both under basal conditions, as well as when cell cycle entry was induced by cdk6 and cyclin D3 overexpression (Fig. 1D,E).

### Introduction of p27^KIP1^ decreases the proliferative response of human β-cells

Next, we explored whether increased nuclear abundance of p27^kip1^ would decrease the proliferative capacity of human β-cells and mimic the decreased proliferative response seen in β-cells from T2D donors. Figure 2A,B shows that adenoviral expression of p27^kip1^ increased nuclear accumulation of p27^kip1^ in human β-cells to a frequency similar to those observed in β-cells from T2D donors. At 100 moi, 33.5 ± 3.7% of β-cells displayed p27^kip1^ in their nuclei, an abundance similar to that in β-cell from T2D donors (29.1 ± 4.5%) (Fig. 1E). In addition, p27 overexpression reduced the proliferation induced by the expression of cdk6 and cyclin D3 or any combination of G1/S cyclins and cdks (Fig. 2C,D).

### p27^kip1^ silencing enhances the proliferative response of human β-cells

We next asked whether p27^kip1^ silencing would increase the proliferative response of human β-cells. As shown in Fig. 3A,B and as expected, p27^kip1^ expression in whole islets increased when proliferation was induced with cdk6 and cyclin D3. Silencing p27^kip1^ by adenoviral delivery of an shRNA targeting p27^kip1^ (shp27^kip1^) significantly decreased the expression of p27^kip1^, both under basal conditions and with the induction of proliferation with cdk6 and cyclin D3. Importantly, the proliferative response induced by cdk6 and cyclin D3 was significantly increased when p27^kip1^ was silenced (Fig. 3C,D). No significant increase in DNA damage response, measured by γP-H2AX immunolabelling, was detected (Fig. 3E,F).

### Skp2 reduces p27^kip1^ expression and enhances the proliferative capacity of human β-cells

Since p27^kip1^ expression is regulated at the protein level by ubiquitinylation and proteosomal degradation^30^,^31^, and since silencing p27^kip1^ increased the proliferative response of human β-cells, we explored whether accelerating p27^kip1^ degradation would lead to increased proliferative response as well. As shown in Fig. 4A, the adenoviral delivery of Skp2 led to a marked increasing expression of Skp2 in human β-cell nuclei. In parallel, the expression of p27^kip1^ was increased when cdk6 and cyclin D3 were overexpressed (Fig. 4B,C). p27^kip1^ expression was reduced when Skp2 was overexpressed in addition to cdk6 and cyclin D3 (Fig. 4B,C). Overexpression of Skp2 alone did not lead to a significant cell cycle entry, as assessed by Ki67 immunolabelling (Fig. 4D,E). However, Skp2 expression significantly enhanced the proliferative response by cdk6 and cyclin D3 (Fig. 4D,E). The DNA damage response was not significantly increased (Fig. 4F,G).

### Skp2, but not p27^kip1^-silencing, induces the expansion of human β-cells

While it is possible to demonstrate increases in markers of β-cell proliferation by downregulating p27^kip1^, it is crucial to determine whether this translates into increased numbers of human β-cells. Thus, we directly counted human β-cell number. As shown in Fig. 5A,B, the expression of Skp2 led to the appearance of greater numbers of β-cells. Surprisingly, however, silencing p27^kip1^ did not.

### Skp2 induces c-Myc transcriptional activity

Since the overexpression of Skp2, but not the silencing of p27^kip1^, led to the expansion of human β-cells, we hypothesized that Skp2 might activate additional pathways besides the downregulation of p27^kip1^. While p27^kip1^ remains a major substrate of Skp2, other targets of Skp2 ubiquitinylation have been described^32^. Specifically, the transcriptional activity of c-Myc is stimulated by Skp2 monoubiquitinylation^25^,^26^,^27^. c-Myc has also been shown to be essential for the development and expansion of rodent insulinoma cells^33^, and modest overexpression of c-Myc, or its induction by small molecules such as harmine, are capable of inducing cell cycle entry of human β-cells^33^,^34^. Thus, we explored whether the induction of cell cycle entry and expansion by Skp2 was associated with the induction of c-Myc target genes in human β-cells. We measured the activation of cell cycle genes, known to be target genes of c-Myc transcriptional activity, and controls, inducing the D-, A, E- and B-cyclins, cdks 1, 2, 4 and 6, cdc25a, E2Fs 1, 2 and 3, p15^Ink4^, p21^cip1^, p27^kip1^, p57^kip2^, Skp2 and c-Myc under control conditions (CTL), or when cdk6 and cyclin D3 were overexpressed with or without Skp2. As a positive control for c-Myc effects, we also measured the expression of the same genes when cdk6, cyclin D3 and c-Myc were overexpressed. As shown in Fig. 6, overexpression of cdk6 and cyclin D3 led to the upregulation of mRNA encoding cyclin A1 (CCNA1), cyclin E1 (CCNE1), cyclin E2 (CCNE2), E2F1 (E2F1), E2F3 (E2F3) and, as expected, cdk6 (CDK6) and cyclin D3 (CCND3). The cell cycle inhibitor, p57 (CDKN1C), was however, significantly downregulated. When Skp2 was overexpressed, cyclin E1, cyclin E2, cdc25a, E2F1 and E2F3 mRNAs were significantly further upregulated. The same mRNAs were further upregulated when c-Myc was overexpressed and their abundance was comparable to that induced by Skp2. E2F2 was the only transcript to be markedly upregulated by c-Myc but not by Skp2 cdk6 or cyclin D3.

To determine whether the upregulation of cyclin E1, cyclin E2, cdc25a, E2F1 and E2F3 by Skp2, cdk6 and cyclin D3 was dependent on c-Myc transcriptional activation, we measured their expression in presence of the c-Myc inhibitor, 1RH. As shown in Fig. 7, the Skp2-induced expression of cyclin E1, cyclin E2, cdc25a, E2F1 and E2F3 were all significantly reduced in presence of 1RH (Fig. 7, left panels). Similarly, the expression of the same genes was also significantly reduced with 1RH when c-Myc was overexpressed, with the sole exception being E2F2. Finally, as expected, the overexpression of cdk6 or cyclin D3 were not affected by the 1RH treatment.

### Skp2 enhances the proliferative response of human β-cells in a c-Myc-dependent manner and increases the proliferative capacity of T2D β-cells

Since Skp2 induces the expression of cell cycle genes in a-c-Myc dependent manner, we wondered whether the proliferative effects of Skp2 were also c-Myc-dependent. As shown in Fig. 8A,B, Skp2 overexpression increased the proliferative response in human β-cells induced to cdk6 and cyclin D3. In presence of 1RH, this effect of Skp2 was markedly attenuated, but the proliferative response to cdk6 and cyclin D3 remained constant. When Skp2 was overexpressed in β-cells from T2D donors, the proliferative response almost doubled (3.7±1.4% to 8.1±1.0% for cdk6 + D3 and cdk6 + D3 + Skp2, respectively) (Fig. 8C).

## Discussion

In this report, we suggest that the proliferative response of β-cells from human T2D subjects is dramatically impaired. We show that this altered proliferative response is accompanied by substantially increased nuclear abundance of the cell cycle inhibitor p27^kip1^ and that increasing nuclear abundance of p27^kip1^ decreases the proliferative response of normal β-cells from non-diabetic donors. Unexpectedly, we show that while both p27^kip1^ gene silencing and downregulation by Skp2 overexpression increase the proliferative response of human β-cells, only Skp2 is capable of inducing significant measurable human β-cell expansion. Skp2 was also able to double the proliferative response of T2D β-cells. We, thus, define c-Myc as a principal Skp2 target for the induction of cell cycle entry and expansion in human β-cells (Fig. 8D).

Evidence indicates that the inadequate functional β-cell mass is an essential component of T2D in humans. Autopsies from normal and T2D subjects show that the β-cell mass is reduced by about 50–65% in obese or lean T2Ds as compared to normal subjects^8^,^9^,^35^,^36^. The β-cell mass is also diminished, to a less extend, in patients with impaired fasting glucose^8^,^35^. Developmental differences, failure of adaptation and increased loss of β-cells via β-cell death or de-differentiation have emerged as the possible causes of this reduced β-cell mass. In rodents, β-cell proliferation contributes to the expansion of β-cell mass in non-diabetic obesity, and a failure to proliferate and/or an increased apoptosis seem to be the features of T2D^1^,^2^,^3^. In humans, data suggest that the proliferation in response to obesity is more limited or absent. Studies by Butler *et al.* show no difference in the very low rates of β-cell proliferation among obese, lean and T2D individuals^8^,^37^. In contrast, a more recent study by Hanley *et al.*, reports increased β-cell proliferation in obese non diabetic subjects as compared to lean non diabetics, with a significant decrease rates of replication in T2D donors^38^. Surprisingly, the group of Kulkarni *et al.* shows an increased proliferation rate in β-cells from T2Ds, as assessed by PCNA immunolabelling, together with the reduced expression of key cell cycle drivers, such as cdk2^39^. However, no difference between the two groups was detected when the proliferation was detected using Ki67 immunolabelling in this study^39^. It is unclear whether this increased PCNA labeling was representative of a true replication or representative of an increased DNA damage, as reported in other cell types^40^.

In our study, we observe that β-cells from T2D donors have a significantly reduced proliferative response. Whether this reduced proliferative response may have contributed to developmental differences and/or failure to adapt later in life in response to an increased metabolic load, or whether this reduced proliferative response was the result of diabetes *per se* is not certain. Nonetheless, in the light of our findings, therapies aiming to regenerate endogenous T2D β-cells appear more challenging than anticipated.

One possible explanation for the reduced proliferative capacity in T2D human β-cells is the increased expression of p27^kip1^, notably in the nuclei of β-cells. Numerous studies in mice indicate that p27^kip1^ is a key regulator of β-cell mass. p27^kip1^-deficient mice display enhanced glucose tolerance, β-cell mass and proliferation^19^. In addition, p27^kip1^ accumulates in terminally differentiated non-proliferative β-cells^19^. Overexpression of p27^kip1^ during development leads to severe glucose intolerance, reduced β-cell mass and proliferation^17^. In mouse models of T2D, loss of p27^kip1^ reduces hyperglycemia by increasing β-cell mass and maintaining hyperinsulinemia, predominantly via β-cell proliferation^15^. Finally, inactivation of the Men1 gene acutely enhances proliferation of pancreatic islet cells, due to loss of p27^kip1^ and p18^18^. Taken together, these studies suggest that p27^kip1^ is a potent cell cycle inhibitor in rodent β-cells.

We have shown previously that the induction of proliferation of normal human β-cells was associated with an increased nuclear translocation of p27^kip1^ and that this nuclear p27^kip1^ was never observed in proliferating β-cells^23^. This suggests that p27^kip1^ may act as an inhibitor of cell cycle in human β-cells, but its precise role has not been explored previously. Here, we show that the level of nuclear p27^kip1^ is already increased under basal conditions in T2D β-cells and is dramatically further enhanced when cell cycle is activated (Fig. 1C,D). A recent study by Chen *et al.* shows an increased level of p27^kip1^ mRNA in pancreatic islets from T2D subjects^41^. We also show that overexpressing p27^kip1^ in normal β-cells to induce levels of nuclear p27^kip1^ similar to those detected in T2D β-cells reduces the proliferative response of human β-cells, mimicking the reduced proliferative response of T2D β-cells (Fig. 2). Altogether, these suggest that p27^kip1^ is a potent inhibitor of cell cycle progression in human β-cells, and may contribute, at least in part, to the decreased proliferative response observed in T2D human β-cells.

Although p27^kip1^ silencing with shRNA or its downregulation by Skp2 both led to a similar increased proliferative response in human β-cells, only Skp2 overexpression resulted in a measurable expansion of human β-cells *in vitro*. This was surprising because studies described above in rodents and humans suggest that p27^kip1^ downregulation is important for a normal progression of β-cell cycle, and that Skp2-mediated p27^kip1^ proteolysis is the principal regulator of p27^kip1^ in β-cells in mice. Like p27^kip1^, Skp2 is essential for β-cell development, replication and β-cell mass^31^. Skp2 knockout mice display accumulation of p27^kip1^ in β-cell nuclei. As a result, Skp2-null mice have a decreased β-cell mass, hypoinsulinemia and glucose intolerance. When challenged with a high fat regimen, Skp2 null-mice become diabetic relative to wild-type littermate, due to their inability to expand β-cell mass in response to a metabolic load^31^. In human islets, we show that expression of Skp2 and p27^kip1^ expressions are inversely correlated as expected. However, the finding that Skp2 overexpression, but not p27 silencing, induces human β-cell proliferation and expansion, suggests that Skp2 operates via an alternative and/or additional pathway beyond p27^kip1^ downregulation. In this regard, it was previously reported that Skp2 transcriptionally activates c-Myc via mono-ubiquitination, which then leads to the upregulation of a subset of target genes involved in the cell cycle entry and completion^25^,^26^,^27^. For example, in HepG2 human hepatoma cells, Skp2 induces cell cycle progression via the activation of c-Myc transcriptional activity and independently of p27^kip1^ expression^42^. In addition, c-Myc has been shown to be associated with β-cell proliferation. In rodent insulinoma cell lines, c-Myc is uniquely and specifically upregulated, and is responsible in major part of their replications^33^. C-Myc is induced by a factor of 1.5 to 3 fold in response to glucose or partial pancreatectomy in association with rodent β-cell proliferation^43^,^44^. As another example, the induction of c-Myc by adenoviral delivery or harmine treatment drives human β-cell replication^33^,^34^. In the present study, the observation that Skp2 induces the transcription of cell cycle mRNA in a c-Myc-depend manner (Figs 6 and 7) supports that Skp2 functions as a transcriptional activator of c-Myc rather than as a component of the SCF complex responsible for p27^kip1^ degradation. Thus, we define c-Myc as an important target for Skp2 in expansion of human β-cells. However, whether Skp2 directly, or indirectly, activates c-Myc transcriptional activity (as shown by multiple arrows and a question mark in Fig. 8C) will require further investigation.

Previous studies have shown that high levels of c-Myc expression lead to immediate cell death and/or dedifferentiation, not only in β-cells, but also other cell types^33^,^44^,^45^. In our hands, however, we did not detect any DNA damage when Skp2 was overexpressed and c-Myc transcriptional activity was induced. We presume that since the induction of c-Myc activity remained moderate, it did not induce cell death, as in other studies using low levels of c-Myc overexpression or harmine in human β-cells have shown that a modest induction of c-Myc by adenoviral delivery or harmine^33^,^34^. In addition, recent studies suggest that the domain-specific ubiquitination of c-Myc by Skp2 control, not only its transcriptional activity, but also its apoptotic activity as well^27^. In that context, the overexpression of Skp2 is capable of inhibiting the recruitment of the tumor suppressor ARF to c-Myc transcriptional domains, leading to the inhibition of c-Myc-induced apoptosis^27^. Thus, one can speculate that, in human β-cells, and in our hands, Skp2, not only drives c-Myc transcriptional activity and cell cycle progression, but also simultaneously inhibits c-Myc induced apoptosis via ARF inhibition. Further studies will determine whether this scenario occurs.

The findings reported here suggest that human β-cells from human T2D organ donors fail to replicate at appropriate levels, and that this failure might contribute to the reduced β-cell mass in people with T2D. We show that increased nuclear abundance of p27^kip1^ is one possible culprit driving this reduced proliferative response. We propose that activating the Skp2-c-Myc pathway may be capable of restoring an appropriate proliferative response leading to β-cell expansion through reduction of p27 combined with direct Skp2 effects on c-Myc activation. Thus, therapies aiming at inducing the Skp2-c-Myc axis in β-cells may be beneficial for β-cells and their expansion in T2D.

## Additional Information

**How to cite this article**: Tiwari, S. *et al.* Definition of a Skp2-c-Myc Pathway to Expand Human Beta-cells. *Sci. Rep.* **6**, 28461; doi: 10.1038/srep28461 (2016).

## Supplementary Material

Supplementary Information
