# Supplementary material for: Definition of a Skp2-c-Myc Pathway to Expand Human Beta-cells
Source: Sci Rep. 2016 Jul 6;6:28461. doi: 10.1038/srep28461 (PMC4933882; doi:10.1038/srep28461)
Supplement: Supplementary Information [file srep28461-s1.pdf]

Definition of a Skp2-c-Myc Pathway to Expand Human Beta-cells.

Running Title: Skp2 and c-Myc in Human β-cells

Shiwani Tiwari MS[1], Chris Roel BS[1], Mansoor Tanwir MD[2], Rachel Wills BS[2], Nidhi Perianayagam[1], Peng Wang PhD[1], and Nathalie M. Fiaschi-Taesch PhD[1,2]

Supplemental Table 1: Primary antisera employed.

Protein/ Antibody	Immunohistochemistry/PLA			Immunoblot		
	Company	Cat#	dilution	Company	Cat#	dilution
**Ki67**	Neomarker	RM-9106-S	1:100	---	---	---
**p27**	Cell Signaling	2552	1:100	Cell Signaling	2552	1:1000
**Skp2**	Cell Signaling	L70	1:100	Cell Signaling	L70	1:500
**Cyclin D3**	---	---	---	Abcam	DCS22	1:500
**Cdk6**	---	---	---	Abcam	Ab-3126	1:500
**P-H2AX**	Millipore	05-636	1:100	---	---	---
**E-Cadherin**	Cell Signaling	3195S	1:100	---	---	---
**Insulin**	DAKO	A0564	1:1000	---	---	---
**Tubulin**	---	---	---	calbiochem	Cp-06	1:2000

Primers		Forward	Reverse
Cyclin D1	CCND1	CAATGACCCCGCACGATTTC	CATGGAGGGCGGATTGGAA
Cyclin D2	CCND2	TTTGCCATGTACCCACCGTC	AGGGCATCACAAGTGAGCG
Cyclin D3	CCND3	TACCCGCCATCCATGATCG	AGGCAGTCCACTTCAGTGC
Cyclin E1	CCNE1	ACTCAACGTGCAAGCCTCG	GCTCAAGAAAGTGCTGATCCC
Cyclin E2	CCNE2	TCAAGACGAAGTAGCCGTTTAC	TGACATCCTGGGTAGTTTTCCTC
Cyclin A1	CCNA1	GAGGTCCCGATGCTTGTCAG	GTTAGCAGCCCTAGCACTGTC
Cyclin A2	CCNA2	GGATGGTAGTTTTGAGTCACCAC	CACGAGGATAGCTCTCATACTGT
Cyclin B1	CCNB1	AATAAGGCGAAGATCAACATGGC	TTTGTTACCAATGTCCCCAAGAG
cdk 1	CDC2	GGATGTGCTTATGCAGGATTCC	CATGTACTGACCAGGAGGGATAG
cdk 2	CDK2	GTACCTCCCCTGGATGAAGAT	CGAAATCCGCTTGTTAGGGTC
cdk 4	CDK4	TCAGCACAGTTCGTGAGGTG	GTCCATCAGCCGGACAACAT
cdk 6	CDK6	CCAGATGGCTCTAACCTCAGT	AACTTCCACGAAAAAGAGGCTT
cdc25a	cdc25a	GTGAAGGCGCTATTTGGCG	TGGTTGCTCATAATCACTGCC
E2F1	E2F1	CATCCCAGGAGGTCACTTCTG	GACAACAGCGGTTCTTGCTC
E2F2	E2F2	CGTCCCTGAGTTCCCAACC	GCGAAGTGTCATACCGAGTCTT
E2F3	E2F3	AGAAAGCGGTCATCAGTACCT	TGGACTTCGTAGTGCAGCTCT
p21	CDKN1B	TAATTGGGGCTCCGGCTAACT	TGCAGGTCGCTTCCTTATTCC
p27	CDKN1A	CGATGGAACTTCGACTTTGTCA	GCACAAGGGTACAAGACAGTG
c- MYC	c-MYC	CCACACATCAGCACAACTACG	CAGCAGGATAGTCCTTCCGAG
skp2	skp2	ATGCCCCAATCTTGTCCATCT	CACCGACTGAGTGATAGGTGT
P15	CDKN2B	CGTTAAGTT TACGGCCAACG	GGTGAGAGTGGCAGGGTCT
p57	CDKN1C	GCGGCGATCAAGAAGCTGT	GCTTGGCGAAGAAATCGGAGA
Actin	ACTB	CATGTACGTTGCTATCCAGGC	CTCCTTAATGTCACGCACGAT

Supplemental Table 2: Primers sequences used for the Real-time PCR

Definition of a Skp2-c-Myc Pathway to Expand Human Beta-cells.

Running Title: Skp2 and c-Myc in Human β-cells

Shiwani Tiwari MS[1], Chris Roel BS[1], Mansoor Tanwir MD[2], Rachel Wills BS[2], Nidhi Perianayagam[1], Peng Wang PhD[1], and Nathalie M. Fiaschi-Taesch PhD[1,2]
